# Activation of bone marrow-derived dendritic cells and CD4^+^ T cell differentiation by outer membrane vesicles of periodontal pathogens

**DOI:** 10.1080/20002297.2022.2123550

**Published:** 2022-09-14

**Authors:** Younggap Lim, Hyun Young Kim, Sun-Jin An, Bong-Kyu Choi

**Affiliations:** Department of Oral Microbiology and Immunology, School of Dentistry, Seoul National University, Seoul, Republic of Korea

**Keywords:** Outer membrane vesicles, periodontitis, red complex, dendritic cells, Th1, Th17

## Abstract

Outer membrane vesicles (OMVs) released from gram-negative bacteria harbor diverse molecules to communicate with host cells. In this study, we evaluated the OMVs of periodontal pathogens for their effects on the activation of dendritic cells and CD4^+^ T cell differentiation. OMVs of *Porphyromonas gingivalis* ATCC 33277, *Treponema denticola* ATCC 33521, and *Tannerella forsythia* ATCC 43037 (‘red complex’ pathogens) were isolated by density gradient ultracentrifugation. Mouse bone marrow-derived dendritic cells (BMDCs) were treated with OMVs, and OMV-primed BMDCs were cocultured with naïve CD4^+^ T cells to analyze the polarization of effector helper T cells. The OMVs upregulated maturation markers, including MHC class II, CD80, CD86, and CD40, on BMDCs. OMVs of *P. gingivalis* and *T. forsythia* induced the expression of the proinflammatory cytokines IL-1β, IL-6, IL-23, and IL-12p70 in BMDCs. In *T. denticola* OMV-primed BMDCs, proinflammatory cytokines were poorly detected, which may be attributed to posttranslational degradation due to the highly proteolytic nature of OMVs. In cocultures of naïve CD4^+^ T cells with OMV-primed BMDCs, OMVs of *P. gingivalis* and *T. denticola* induced the differentiation of Th17 cells, whereas *T. forsythia* OMVs induced Th1 cell differentiation. These results demonstrate that OMVs derived from the ‘red complex’ periodontal pathogens induce maturation of BMDCs and differentiation of naïve CD4^+^ T cells to Th1 or Th17 cells.

## Introduction

Periodontitis is a chronic inflammatory disease characterized by periodontal tissue destruction, often leading to tooth loss [1]. The proinflammatory response of periodontal tissue is induced by oral microbiota dysbiosis in the subgingival biofilm, with an increased proportion of the ‘red complex’ bacteria *Porphyromonas gingivalis, Treponema denticola*, and *Tannerella forsythia* [2]. The ‘red complex’ is highly associated with periodontal lesions and disrupts host immune systems by producing proteases, such as gingipains and dentilisin [2]. In addition, periodontal pathogen-derived molecules and bacteria themselves are related to the pathogenesis of systemic diseases, such as Alzheimer’s diseases, cardiovascular disease, diabetes, and rheumatoid arthritis [3,4].

Extracellular vesicles (EVs) are nanosized with a diameter of 30–2,000 nm and enclosed by a lipid bilayer membrane [5]. EVs are secreted by all living cells and contain various biomolecules, such as proteins, lipids, nucleic acids, and metabolites, that can mediate cell–cell communication and change the physiological status of recipient cells [5]. EVs named outer membrane vesicles (OMVs) are released by gram-negative bacteria and those named membrane vesicles (MVs) are released by gram-positive bacteria [6]. Since bacterial EVs contain various microbe-associated molecular patterns (MAMPs) and virulence factors, bacterial EVs can stimulate host cells to induce inflammatory responses [7]. Innate immune responses to EVs derived from periodontal pathogens have been reported. OMVs of *P. gingivalis, T. denticola*, and *T. forsythia* induce Toll-like receptor (TLR) signaling pathways and inflammasome activation [8,9]. In addition, bacterial EVs can spread to host tissues through the bloodstream and induce local and systemic inflammatory responses [10]. Recently, it has been reported that EVs derived from periodontal pathogens are responsible for periodontitis-associated systemic diseases [11–13].

Dendritic cells (DCs) are professional antigen-presenting cells (APCs) that take up and process antigens and then present them to T cells to initiate proper adaptive immunity [14]. APCs express various pattern-recognition receptors (PRRs), which recognize MAMPs to trigger innate immune responses against invading pathogens. DCs are matured by PRR signaling and then migrate to draining lymph nodes to present antigens to naïve T cells. According to the cytokine milieu, naïve CD4^+^ T cells differentiate into a distinct lineage of helper T cell subsets to modulate immune responses in the appropriate direction [15]. CD4^+^ T cells in gingival tissue play a central role in orchestrating oral mucosal immunity [16]. In a healthy state, mechanical barrier damage caused by mastication induces Th17 cells for protective immunity of the oral mucosa [17]. However, when the burden of dysbiotic oral bacteria is increased, dysregulation of Th17 cell responses is induced, which leads to chronic inflammation, eventually developing into periodontal disease and bone resorption [18,19].

*P. gingivalis* OMVs induce neutrophil infiltration [20] and foam cell formation in the presence of low-density lipoproteins [21]. EVs of *Filifactor alocis*, a gram-positive periodontal pathogen, induce systemic bone loss through TLR2-mediated osteoclastogenesis [13]. OMVs of *Aggregatibacter actinomycetemcomitans*, a gram-negative periodontal pathogen, can cause neuroinflammation through activation of brain monocytes and microglial cells [22]. However, the OMVs of periodontal pathogens have not been studied for their effects on DC activation and CD4^+^ T cell differentiation. In this study, we analyzed the maturation of DCs and the mode of helper T cell differentiation induced by OMVs derived from the ‘red complex’ bacteria *P. gingivalis* ATCC 33277, *T. denticola* ATCC 33521, and *T. forsythia* ATCC 43037 using mouse bone marrow-derived dendritic cells (BMDCs) and splenic naïve CD4^+^ T cells.

## Materials and methods

### Bacteria

*P. gingivalis* ATCC 33277 was cultured in brain heart infusion (BHI; BD Biosciences, San Jose, CA, USA) broth supplemented with 5 μg/ml hemin (Sigma-Aldrich, St. Louis, MO, USA) and 1 μg/ml vitamin K_3_ (menadione; Sigma-Aldrich) under anaerobic conditions (10% CO_2_, 10% H_2_, and 80% N_2_) at 37°C for 48 h. *T. denticola* ATCC 33521 and *T. forsythia* ATCC 43037 were cultured in new oral spirochete broth (NOS; ATCC medium 1494) under anaerobic conditions at 37°C for 60 and 48 h, respectively. For *T. forsythia*, 5 μg/ml hemin, 1 μg/ml vitamin K_3_, and 10 μg/ml *N*-acetylmuramic acid (NAM; Sigma-Aldrich) were added.

### Isolation of outer membrane vesicles (OMVs)

OMVs were isolated as previously described with some modification [13]. Briefly, 400 ml of each bacterial culture supernatant was collected and centrifuged at 10,000 × *g* and 4°C for 10 min. Then, cell-free culture supernatants were filtered using a 0.2 μm polyethersulfone membrane filter system (Corning, New York, NY, USA). The filtered culture supernatants were subjected to ultracentrifugation at 120,000 × *g* and 4°C for 3 h. The supernatants were discarded, and the pellets were resuspended in sterile phosphate-buffered saline (PBS; Welgene, Daegu, Korea) followed by ultracentrifugation at 120,000 × *g* and 4°C for 2 h. The pellets were resuspended in PBS and mixed with 60% iodixanol solution (OptiPrep^TM^; Sigma-Aldrich) to obtain a 40% solution. The iodixanol-diluted pellets were laid on the bottom of an ultracentrifuge tube, and 35%, 30%, 25%, 20%, and 5% iodixanol solution diluted with PBS was overlaid. The discontinuous density gradient layers were ultracentrifuged at 100,000 × *g* and 4°C for 18 h. The same volume (1.3 ml) of each fraction was harvested from top to bottom. The nanoparticle concentrations in each fraction were analyzed via nanoparticle tracking analysis (NTA) as described below. The nanoparticle-enriched fractions were mixed with PBS (10 ml) and ultracentrifuged at 120,000 × *g* and 4°C for 2 h. After removal of the supernatant, the OMV pellets were resuspended in PBS. The protein concentration of OMVs was determined by bicinchoninic acid (BCA) assays (Thermo Fisher Scientific Inc., Waltham, MA, USA) according to the manufacturer’s instructions. The particle number in 10 μg proteins of each OMV was as follow: *P. gingivalis* OMVs (1.51 ± 0.54 × 10^10^), *T. forsythia* OMVs (1.39 ± 0.40 × 10^10^), and *T denticola* OMVs (3.83 ± 0.33 × 10^10^). The isolated OMVs were aliquoted and stored at −80°C until use.

### Transmission electron microscopy (TEM)

Five microliters of OMVs were loaded onto a glow-discharged formvar/carbon-coated copper grid (Electron Microscopy Sciences, Hatfield, PA, USA) for 1 min, washed twice with distilled water, and then stained with 2% uranyl acetate (Electron Microscopy Sciences) for 1 min. The negatively stained OMVs were imaged via TEM (LIBRA 120; Carl Zeiss, Jena, Germany) at 120 kV.

### Nanoparticle Tracking Analysis (NTA)

The size and concentration of OMVs were measured with a NanoSight LM10 instrument (Malvern Instruments Ltd., Worcestershire, UK). Each OMV was diluted in nanoparticle-free PBS to adjust the proper concentration range (5 × 10^8^ – 2 × 10^9^ particles/ml). NTA software (Ver. 2.5, Malvern Instruments Ltd.) was used for data analysis, and the acquisition settings used in this experiment were as follows: screen gain, 12; camera level, 15; and detection threshold, 3.

### Mice

Eight-week-old C57BL/6N mice were purchased from Orient Bio (Seongnam, Korea). All mouse experiments were approved by the Institutional Animal Care and Use Committee of Seoul National University (SNU-210602-1).

### Bone marrow-derived dendritic cells (BMDCs)

Bone marrow cells were isolated from the femur and tibia of eight-week-old C57BL/6N mice. The isolated bone marrow cells were cultured in RPMI 1640 (Welgene) supplemented with 10% heat-inactivated fetal bovine serum (FBS; HyClone Laboratories, Inc., Logan, UT, USA), 1% penicillin/streptomycin (Gibco, Waltham, MA, USA), 20 ng/ml recombinant murine granulocyte-macrophage colony-stimulating factor (rmGM-CSF; Peprotech, Rocky Hill, NJ, USA), and 55 μM β-mercaptoethanol (Gibco) for 7 days (37°C, 5% CO_2_). On Day 3, fresh medium was added. On Day 7, floating cells were harvested and labeled with biotin anti-CD11c antibody (BioLegend, San Diego, CA, USA) and subsequently incubated with anti-biotin microbeads (Miltenyi Biotec, Bergisch Gladbach, Germany). The CD11c^+^ cells were isolated using an MS column (Miltenyi Biotec) according to the manufacturer’s instructions. The isolated CD11c^+^ BMDCs were stimulated with 10 μg/ml periodontal pathogen OMVs in RPMI 1640 complete medium (supplemented with 10% heat-inactivated FBS and 1% penicillin/streptomycin) for 24 h, and the expression of cell surface markers and cytokines was analyzed via flow cytometry and ELISA, respectively.

### Coculture of naïve CD4^+^ T cells with CD11c^+^ BMDCs

Naïve CD4^+^ T cells were isolated from the spleen of eight-week-old C57BL/6N mice using a naïve CD4^+^ T cell isolation kit (STEMCELL Technologies, Vancouver, BC, Canada). CD11c^+^ BMDCs were stimulated with 10 μg/ml OMVs of periodontal pathogens in RPMI 1640 complete medium for 5 h and then washed with sterile PBS. OMV-primed BMDCs were cocultured with naïve CD4^+^ T cells (DCs:T cells = 1:5) in the presence of 55 μM β-mercaptoethanol and 0.3 ng/ml anti-CD3ε antibody (clone 145–2C11; Bio X Cell, West Lebanon, NH, USA) in RPMI 1640 complete medium for 4 days (37°C, 5% CO_2_). For neutralization of cytokines, 10 μg/ml anti-mouse-IL-6 antibody (clone MP5-20F3; isotype: rat IgG1; Bio X Cell) and anti-mouse-IL-12p40 antibody (clone C17.8; isotype: rat IgG2a; Bio X Cell) were used.

### Flow cytometry

The Fc receptors on CD11c^+^ BMDCs were blocked with TruStain FcX™ PLUS (anti-mouse CD16/32; BioLegend). The surface molecules of BMDCs were stained with FITC anti-mouse I-A/I-E antibody (MHC class II; clone M5/114.15.2; isotype: FITC Rat IgG2b; BioLegend), BB700 anti-mouse CD40 antibody (clone 3/23; isotype: BB700 Rat IgG2a; BD Biosciences), APC anti-mouse CD80 antibody (clone 16–10A1; isotype: APC Armenian Hamster IgG; BioLegend), and PE anti-mouse CD86 antibody (clone GL-1; isotype: PE Rat IgG2a; BioLegend).

For intracellular staining of CD4^+^ T cells, the cells were incubated with 50 ng/ml phorbol 12-myristate 13-acetate (Sigma-Aldrich), 1 µM ionomycin (Sigma-Aldrich), and GolgiPlug^TM^ (BD Biosciences) for 5 h. The cells were stained with Ghost Dye^TM^ Violet 510 (Tonbo Biosciences, San Diego, CA, USA) and fixed with Cyto-Fast^TM^ Fix/Perm buffer (BioLegend), followed by washing with Cyto-Fast^TM^ Perm/Wash buffer (BioLegend). The intracellular cytokines were stained with PE anti-mouse IFN-γ antibody (clone XMG1.2; isotype: PE Rat IgG1; BioLegend), Brilliant Violet 421^TM^ anti-mouse IL-4 antibody (clone 11B11; isotype: Brilliant Violet 421™ Rat IgG1; BioLegend), and Alexa Fluor® 647 anti-mouse IL-17A antibody (clone TC11-18H10; isotype: Alexa Fluor® 647 Rat IgG1; BD Biosciences). Surface CD4 was stained with BB700 anti-mouse CD4 antibody (clone RM4-5; isotype: BB700 Rat IgG2a; BD Biosciences). The fluorescence intensity of the cells was measured with a FACS LSRFortessa X-20 (BD Biosciences). The FCS data files were analyzed using FlowJo software version 10.1 (BD Biosciences).

### Enzyme-linked immunosorbent assay (ELISA)

The levels of IL-1β, IL-4, IL-6, IL-23, and IL-12p70 in the culture supernatants of BMDCs stimulated with OMVs of periodontal pathogens were measured using ELISA kits (BioLegend and R&D Systems, Minneapolis, MN, USA) according to the manufacturer’s instructions. The optical density of each well was measured with an Epoch2 microplate reader (BioTek Instruments Inc., Winooski, VT, USA) at wavelengths of 450 nm and 540 nm.

### Quantitative real-time polymerase chain reaction (qRT–PCR)

Total RNA of CD11c^+^ BMDCs was isolated using an easy-BLUE™ total RNA extraction kit (iNtRON Biotechnology, Seongnam, Korea) according to the manufacturer’s instructions. The concentration of total RNA was quantified using a NanoDrop spectrophotometer (Thermo Fisher Scientific). To eliminate genomic DNA contamination, the RNA samples were treated with DNase I (Amplification Grade; Thermo Fisher Scientific) according to the manufacturer’s instructions. Complementary DNA (cDNA) was synthesized in a 30 μl reaction volume using 1 μg of DNase-treated RNA, oligo dT primer (Cosmo Genetech, Seoul, Korea), and an M-MLV reverse transcriptase kit (Promega, Madison, WI, USA) according to the manufacturer’s instructions. For quantitative real-time polymerase chain reaction (qRT–PCR), cDNA (2 μl) was mixed with primer pairs (200 nM each) and 10 μl of Power SYBR® Green Master mix (Applied Biosystems, Waltham, MA, USA) in a 20 μl reaction volume. After an initial denaturation at 95°C for 5 min, cDNA was amplified for 40 cycles of denaturation (95°C, 15s) and annealing (60°C, 1 min) using a StepOne^TM^ Plus real-time PCR system (Applied Biosystems). Gene expression levels determined by qRT–PCR were normalized against glyceraldehyde-3-phosphate dehydrogenase (GAPDH) levels and calculated according to the 2^−ΔΔ*CT*^ method. The primers used in this experiment were as follows: *Gapdh*, forward 5′-AAT GGT GAA GGT CGG TGT GAA-3′ and reverse 5′-CAA TCT CCA CTT TGC CAC TGC-3′; *Il12a*, forward 5′-GAA GAC ATC ACA CGG GAC CAA-3′ and reverse 5′-CCA GGC AAC TCT CGT TCT TGT-3′; *Il23a*, forward 5′-CCA GCG GGA CAT ATG AAT CTA C-3′ and reverse 5′-TGT CCT TGA GTC CTT GTG GG-3′. *Il1b* and *Il6* were used as previously described [23].

### Degradation of proinflammatory cytokines by periodontal pathogen OMVs

In 96-well cell culture plates, 1 ng/ml of recombinant murine IL-1β (R&D Systems), IL-6 (Peprotech), IL-23 (R&D Systems), and IL-12p70 (Peprotech) were incubated with OMVs (1 and 10 μg/ml) in 200 μl/well RPMI 1640 complete medium for 24 h at 37°C in humidified aerobic conditions (5% CO_2_). BMDCs were stimulated with 100 ng/ml Pam3CSK4 for 24 h at 37°C. Culture supernatants of Pam3CSK4-treated BMDCs were incubated with *T. denticola* OMVs in the presence or absence of 2 mM phenylmethylsulfonyl fluoride (PMSF, Thermo Fisher Scientific), a serine protease inhibitor, for 1 h. In addition, *T. denticola* OMVs were heated at 95°C for 10 min and incubated with culture supernatants of Pam3CSK4-treated BMDCs for 1 h. The levels of the remaining cytokines in the medium or culture supernatants were measured by ELISA.

### Statistics

The mean value ± standard deviation (*SD*) was determined for each group. Student’s *t*-test was used to determine the significance of differences between two groups. One-way analysis of variance (ANOVA) with Dunnett’s post-test was used to determine the significance of differences among more than two groups. A *p*-value less than 0.05 was considered statistically significant. All statistical analyses were performed using GraphPad Prism software (GraphPad Software Inc., San Diego, CA, USA).

## Results

### Characterization of OMVs from periodontal pathogens

**Figure 1.**
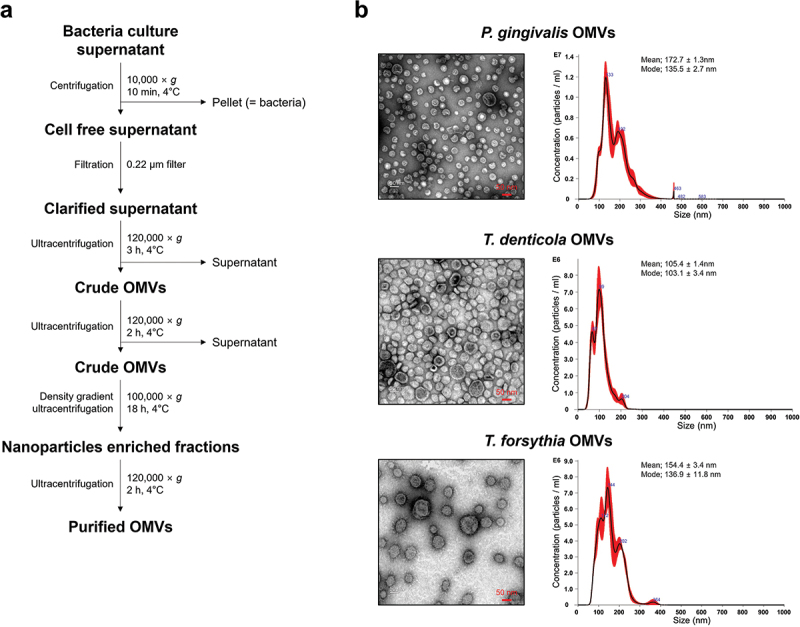
Isolation and characterization of periodontal pathogen OMVs.

OMVs of *P. gingivalis* ATCC 33277, *T. denticola* ATCC 33521, and *T. forsythia* ATCC 43037 were isolated from bacterial culture supernatants by ultracentrifugation combined with iodixanol density gradient ultracentrifugation (Figure 1a). The size and number of OMVs were measured by NTA, which can directly measure the concentration and size of nanoparticles (OMVs) in a sample by light scattering technologies [24]. Approximately 750 μg protein of *P. gingivalis* OMVs, 210 μg protein of *T. denticola* OMVs, and 1200 μg protein of *T. forsythia* OMVs were harvested from 400 ml of each bacterial culture supernatants. The OMVs showed a round and cup-shaped morphology in the negatively stained TEM images, and their sizes ranged from 50 nm to 400 nm (Figure 1b).

(a) Schematic diagram of OMV isolation procedures. (b) The OMVs were negatively stained with uranyl acetate and observed via TEM at 120 kV (left panel) with a magnification of 40,000 × . Scale bars represent 50 nm. The size and concentration of the indicated OMVs were measured by NTA (right panel).

### Maturation of BMDCs induced by OMVs of periodontal pathogens

**Figure 2.**
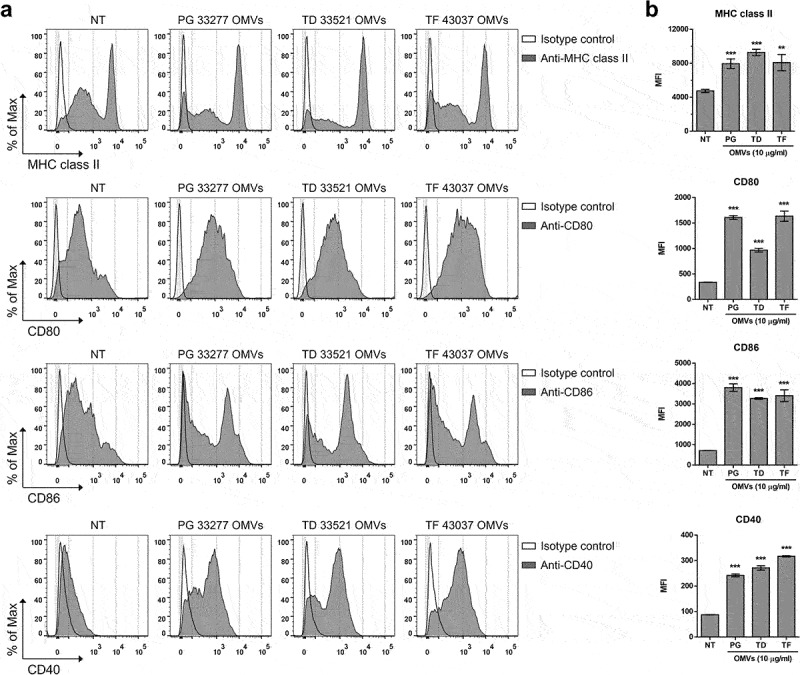
Maturation of BMDCs induced by periodontal pathogen OMVs.

Activation of APCs by microbial components is essential for initiating naïve CD4^+^ T cell differentiation [25]. Activated DCs are mature and express high levels of major histocompatibility complex (MHC) class II and costimulatory molecules, including CD80, CD86, and CD40, on the cell surface to signal to naïve CD4^+^ T cells [25]. To analyze the maturation of BMDCs, they were stimulated with OMVs of periodontal pathogens for 24 h, and the expression levels of MHC class II and costimulatory molecules on the cell surface were analyzed via flow cytometry. All OMVs significantly induced the expression of MHC class II, CD80, CD86, and CD40 (Figure 2a,b). These results suggest that OMVs of periodontal pathogens induced the maturation of BMDCs.

BMDCs were stimulated with 10 μg/ml of the indicated OMVs for 24 h. The expression of MHC class II (I-A/I-E), CD80, CD86, and CD40 on BMDCs was analyzed via flow cytometry. The experiments were performed three times independently, and representative data are shown in the histograms (a) and by the mean fluorescence intensity (MFI) (b). The empty solid lines represent the isotype control. The data are presented as the mean ± *SD* of triplicate assays and were analyzed by one-way ANOVA. ** *p* < 0.01 and *** *p* < 0.001 compared to the control (NT). NT, non-treatment; PG, *P. gingivalis* ATCC 33277; TD, *T. denticola* ATCC 33521; TF, *T. forsythia* ATCC 43037.

### Expression of CD4^+^ T cell polarization cytokines secreted from BMDCs induced by OMVs of periodontal pathogens

**Figure 3.**
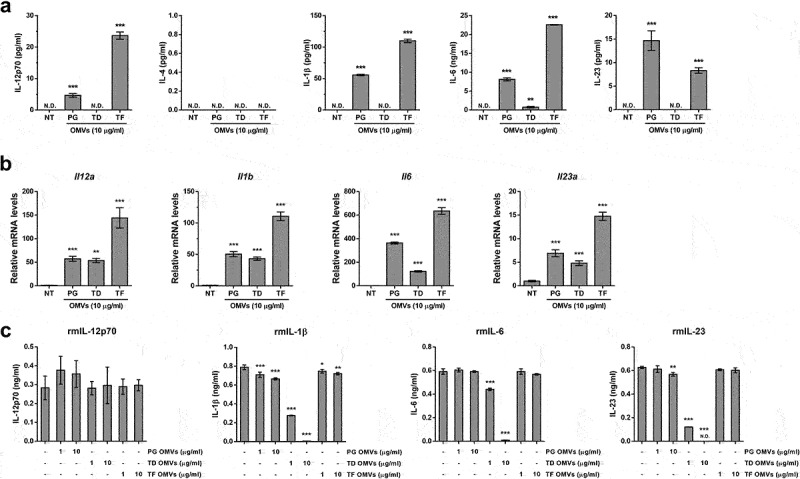
Effects of periodontal pathogen OMVs on proinflammatory cytokine secretion in BMDCs and cytokine degradation.

Since the polarization of naïve CD4^+^ T cells is regulated by cytokines [25], we measured the cytokines released from BMDCs stimulated with OMVs of periodontal pathogens using ELISA. IL-12p70 was measured for Th1 cell polarization, and IL-4 for Th2 cell polarization. IL-1β, IL-6, and IL-23 were measured for Th17 cell polarization. *P. gingivalis* OMVs and *T. forsythia* OMVs significantly induced the secretion of IL-12p70, IL-1β, IL-6, and IL-23 from BMDCs (Figure 3a). *T. denticola* OMVs increased IL-6 but did not induce IL-12p70, IL-1β, or IL-23 secretion. No OMVs induced IL-4 secretion. As *T. denticola* OMVs activated BMDCs as efficiently as other OMVs, as judged by the expression of costimulatory molecules (Figure 2), we speculated that *T. denticola* OMVs might have the ability to degrade cytokines. As expected, *T. denticola* OMVs significantly induced *Il12a, Il1b, Il6*, and *Il23a*, similar to or less than *P. gingivlias* OMVs and *T. forsythia* OMVs (Figure 3b). To examine the proteolytic activity of the OMVs, we incubated recombinant cytokines with the OMVs and measured the remaining cytokines via ELISA. As shown in Figure 3c, *T. denticola* OMVs degraded IL-1β, IL-6, and IL-23 but not IL-12p70 in a dose-dependent manner (Figure 3c). To determine whether *T. denticola* OMVs were able to degrade cytokines secreted from BMDM stimulated with Pam3CSK4, a synthetic lipopeptide known to mimic bacterial lipoproteins, the culture supernatants of BMDCs were harvested and then incubated with *T. denticola* OMVs. *T. denticola* OMVs significantly degraded IL-1β, IL-6, and IL-23 in BMDM culture supernatants (Supplementary Figure S1). Heat- or PMSF-treated *T. denticola* OMVs lost their proteolytic activity against IL-1β, IL-6, and IL-23. These results suggest that *T. denticola* OMVs induced the production of IL-1β, IL-6, and IL-23, which were subsequently degraded. *P. gingivalis* OMVs also degraded IL-1β and IL-23 but to a lesser extent than *T. denticola* OMVs. *T. forsythia* OMVs degraded IL-1β. These results indicate that OMVs of periodontal pathogens are likely to induce Th1 or Th17 polarization and that *T. denticola* OMVs possess strong proteolytic activity compared to other OMVs.

(a) BMDCs were stimulated with 10 μg/ml of the indicated OMVs for 24 h. The expression levels of IL-12p70, IL-4, IL-1β, IL-6, and IL-23 in the culture supernatants were measured with ELISA. (b) BMDCs were stimulated with 10 μg/ml of the indicated OMVs for 3 h. The relative mRNA levels of the indicated genes were analyzed via qRT–PCR. (c) One nanogram per ml of recombinant murine IL-12p70, IL-1β, IL-6, and IL-23 were coincubated with the indicated OMVs at 37°C for 24 h in RPMI 1640 complete medium. The level of the cytokines remaining in the medium was analyzed using ELISA. The experiments were performed three times independently. The data are presented as the mean ± *SD* of triplicate assays and were analyzed by one-way ANOVA. * *p* < 0.05, ** *p* < 0.01, and *** *p* < 0.001 compared to the control (NT). NT, non-treatment; N.D., not detected. PG, *P. gingivalis* ATCC 33277; TD, *T. denticola* ATCC 33521; TF, *T. forsythia* ATCC 43037.

### Th1/Th17 polarization of CD4^+^ T cells by BMDCs stimulated with OMVs of periodontal pathogens

**Figure 4.**
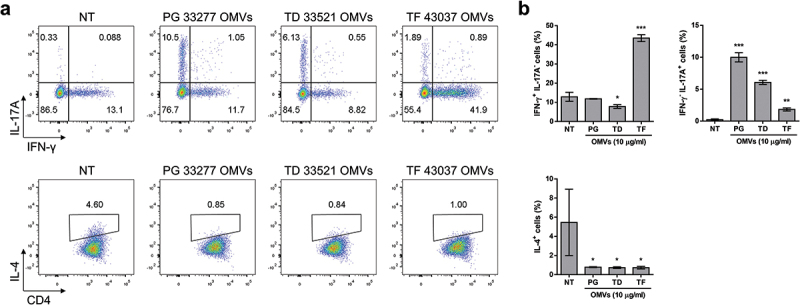
Differentiation of naïve CD4^+^ T cells induced by OMV-primed BMDCs.

Naïve CD4^+^ T cells were cocultured with OMV-primed BMDCs to analyze the effect of OMVs on T cell polarization. To exclude the possibility that OMVs of periodontal pathogens can degrade cytokines from OMV-primed BMDCs, we vigorously washed OMV-primed BMDCs at 5 h posttreatment and then cocultured them with naïve CD4^+^ T cells. BMDCs stimulated with OMVs of *P. gingivalis* and *T. denticola* induced IL-17A^+^ T cells, while *T. forsythia* OMV-primed BMDCs mainly induced IFN-γ^+^ T cells along with some IL-17A^+^ T cells (Figure 4a,b Supplementary Figure S2). IL-4^+^ T cells were not detected in any coculture. These results indicate that OMVs of *P. gingivalis* and *T. denticola* induced Th17 polarization, whereas *T. forsythia* OMVs favored Th1 polarization rather than Th17.

BMDCs were stimulated with 10 μg/ml of the indicated OMVs for 5 h. After three times of washing with PBS, the BMDCs were cocultured with naïve CD4^+^ T cells for 4 days in the presence of soluble anti-CD3ε antibody and analyzed via flow cytometry. The experiments were performed four times independently, and representative data are shown as dot plots (a) and the proportions of intracellular IFN-γ, IL-17A, and IL-4 in CD4^+^ T cells (b). The data are presented as the mean ± *SD* of triplicate assays and were analyzed by one-way ANOVA. * *p* < 0.05, ** *p* < 0.01, and *** *p* < 0.001 compared to the control (NT). NT, non-treatment; PG, *P. gingivalis* ATCC 33277; TD, *T. denticola* ATCC 33521; TF, *T. forsythia* ATCC 43037.

### Evaluation of the cytokines responsible for CD4^+^ T cell differentiation induced by OMVs of periodontal pathogens

**Figure 5.**
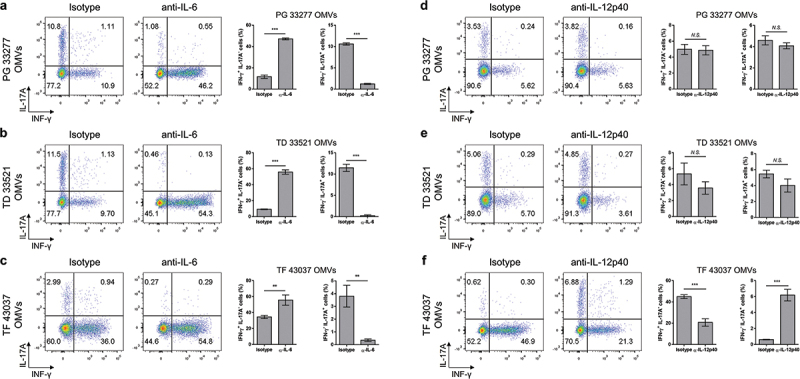
Key cytokines for Th1 and Th17 differentiation induced by OMV-primed BMDCs.

To evaluate which cytokine contributes to the differentiation of Th1 or Th17 cells induced by OMV-primed BMDCs, we treated BMDCs with neutralizing antibodies against IL-12 and IL-6 during coculture and then analyzed CD4^+^ T cell differentiation. Neutralization of IL-6 significantly increased IFN-γ^+^ cells but reduced IL-17A^+^ cells in the coculture of each periodontal pathogen derived OMV-primed BMDC with naïve CD4^+^ T cells (Figure 5a–c). Neutralization of IL-12p40 decreased IFN-γ^+^ cells but increased IL-17A^+^ cells in the coculture of *T. forsythia* OMV-primed BMDCs with naïve CD4^+^ T cells (Figure 5f). However, IFN-γ^+^ and IL-17A^+^ cells were not affected in the coculture of *P. gingivalis* OMV- and *T. denticola* OMV-primed BMDCs with naïve CD4^+^ T cells (Figure 5d,e). These results suggest that IL-6 is major cytokine in Th17 differentiation by OMVs of *P. gingivalis* and *T. denticola*, whereas IL-12 is responsible for Th1 differentiation by *T. forsythia* OMVs.

BMDCs were stimulated with 10 μg/ml *P. gingivalis* OMVs (a and d), *T. denticola* OMVs (b and e), and *T. forsythia* OMVs (c and f) for 5 h. After three times of washing with PBS, the BMDCs were cocultured with naïve CD4^+^ T cells for 4 days in the presence of soluble anti-CD3ε antibody and 10 μg/ml anti-IL-6 (a to c) and anti-IL-12p40 (d to f) antibodies and then analyzed via flow cytometry. Representative data are shown as dot plots (left panel) and the proportion of cells (right panel) expressing intracellular IFN-γ and IL-17A among CD4^+^ T cells. The experiments were performed three times independently. The data are presented as the mean ± *SD* of triplicate assays and were analyzed by Student’s *t*-test. ** *p* < 0.01 and *** *p* < 0.001 compared to the control (isotype). N.S., not significant. PG, *P. gingivalis*; TD, *T. denticola*; TF, *T. forsythia*.

## Discussion

The responses of T lymphocytes to dysbiotic oral bacteria play an essential role in the immunopathogenesis of periodontal disease [26]. As OMVs harbor various immunostimulatory molecules [7], the present study determined the role of the OMVs of periodontal pathogens in helper T cell polarization through DC maturation. The OMVs of periodontal pathogens activated DCs to secrete effector cytokines of Th1 and Th17 cells but not Th2 cells. *P. gingivalis* 33277 OMV- and *T. denticola* 33521 OMV-primed BMDCs induced the differentiation of naïve CD4^+^ T cells into Th17 cells. In contrast, *T. forsythia* 43037 OMV-primed BMDCs favored the polarization of Th1 cells rather than Th17 cells.

The maturation of DCs is essential for linking innate immunity to adaptive immunity [25]. There is some evidence that DC maturation by the OMVs of periodontal pathogens might occur through TLR signaling pathways. First, the ‘red complex’ bacteria induces immune responses mainly through TLR2 and TLR4 [27–29]. Second, the OMVs of periodontal pathogens harbor various MAMPs, such as lipopolysaccharide/lipooligosaccharide (LPS/LOS), lipoproteins, peptidoglycan, DNA, and RNA, which are the ligands of TLRs [8]. Several studies have reported that other bacterial OMVs can induce DC maturation through TLR signaling pathways [30–32]. Furthermore, since OMVs are potent immune stimulators and deliver bacteria-specific antigens without the bacteria being present, OMVs are receiving attention as an ideal vaccine against infectious diseases [33]. Therefore, further studies are needed to identify the molecular mechanisms of TLR signaling that are responsible for DC maturation induced by MAMPs in the periodontal pathogen OMVs.

For normalization of OMV treatment, one must choose either a biomolecule or a number of OMVs. According to Cecil *et al*. (2016), the amounts of protein, LPS, lipoprotein, peptidoglycan, DNA, and RNA were too different in the same number of OMVs of *P. gingivalis, T. denticola*, and *T. forsythia* [8]. In the report, the degree of TLR2 and TLR4 activation by the same amount of OMV proteins was similar to each other, but the degree of TLR2 and TLR4 activation by the same number of OMVs was very different, especially in the case of *T. denticola* OMVs. Although the particle number in 10 μg proteins of *P. gingivalis* OMVs and *T. forsythia* OMVs was similar, their ability to induce Th1 or Th17 differentiation was different. The particle number in 10 μg proteins of *T. denticola* OMVs was higher than that in 10 μg proteins of *P. gingivalis* OMVs and *T. forsythia* OMVs, but showed similar BMDC-activating abilities to *P. gingivalis* OMVs and *T. forsythia* OMVs. Therefore, we believe that protein quantification of OMVs is a reliable criterion for assessing host response to OMVs and that different T cell polarization by each OMVs might be due to their different qualitative rather than quantitative nature.

The present study demonstrated that *P. gingivalis* OMVs induced DC-mediated Th17 polarization. It seems that the ability of *P. gingivalis* OMVs to induce Th17 polarization is similar to that of *P. gingivalis* whole cells. *P. gingivalis* induces Th17 responses *in vivo* and *in vitro* by activating DCs and monocytes [34–37]. *P. gingivalis* LPS has been found to upregulate Th17 cell differentiation from activated human naïve CD4^+^ T cells in the presence of Th17-driven cytokines [38]. Th17 cells induced by *P. gingivalis* affect the pathogenesis of periodontitis and systemic diseases, such as rheumatoid arthritis [39]. *P. gingivalis* oral infection induces alveolar bone resorption and aggravates the severity of arthritis, which may be associated with increased systemic Th17 responses [40,41]. Since *P. gingivalis* OMVs can spread to host tissues through the bloodstream [11], *P. gingivalis* OMVs may induce Th17 differentiation, leading to periodontitis and other systemic diseases. Since only the *P. gingivalis* ATCC 33277 strain was used in this study, it is necessary to analyze various *P. gingivalis* strains, including W50 and gingipain mutant strains, for DC maturation and T cell differentiation.

Unlike *P. gingivalis*, the role of *T. denticola* in CD4^+^ T cell responses has not been well studied. The present study demonstrated for the first time that *T. denticola* OMVs induced DC-mediated Th17 polarization but not Th1. IL-6 released from both *P. gingivalis* OMV- and *T. denticola* OMV-primed BMDCs was found to play a pivotal role in Th17 polarization. Among various cytokines from mature DCs, IL-6 is an essential cytokine for the commitment of Th17 cells [42,43]. IL-6 activates the STAT3 signaling pathway through gp130 on naïve CD4^+^ T cells [42], and STAT3 is a crucial transcription factor for Th17 cell differentiation [43]. In addition, IL-6 inhibits the differentiation of Th1 cells by inhibiting SOCS1 function [44].

In contrast to *P. gingivalis* OMV- and *T. denticola* OMV-primed BMDCs, *T. forsythia* OMV-primed BMDCs preferentially induced the differentiation of Th1 cells rather than Th17 cells. IL-12 was found to play a pivotal role in Th1 differentiation by *T. forsythia* OMV-primed BMDCs. IL-12 is known as an inducer of naïve CD4^+^ T cell differentiation into Th1 cells [45]. *T. forsythia* OMVs significantly induced IL-12p70 expression in BMDCs compared to OMVs of *P. gingivalis* and *T. denticola*. Indeed, the neutralization of IL-12 reduced Th1 cells but increased Th17 cells in the coculture of *T. forsythia* OMV-primed BMDCs with naïve CD4^+^ T cells. Furthermore, the S-layer glycan of *T. forsythia* was previously reported to restrain Th17 cell responses in a mouse model and human peripheral blood mononuclear cell study [46,47]. The S-layer glycan is a typical structure of *T. forsythia* and protects the bacterium from recognition by DCs [46]. Further studies are needed to identify the detailed molecular mechanisms underlying the ability of OMVs from different bacterial species to differentially regulate T cell polarization.

Among the three periodontal pathogen OMVs, *T. denticola* OMVs showed the highest proteolytic activity. Dentilisin is a chymotrypsin-like proteinase located in the outer membrane of *T. denticola* [48]. Veith *et al*. showed that dentilisin is one of the abundant proteins in *T. denticola* OMVs, and its topology on *T. denticola* OMVs faces the extracellular space [49]. It has been shown that dentilisin can degrade several host proteins, including proinflammatory cytokines, such as IL-1β, IL-6, IL-8, and TNF-α [50]. Ginginpains are well-characterized proteolytic enzymes and virulence factors of *P. gingivalis* [51]. There are three types of gingipains. RgpA and RgpB are arginine-specific cysteine proteinases, and Kgp is a lysine-specific cysteine proteinase [52]. Gingipains are abundant on *P. gingivalis* OMVs [53] and contribute to evasion of the host immune surveillance system by degrading cytokines such as IL-1β, IL-6, and IFN-γ [54]. In this study, *P. gingivalis* OMVs degraded recombinant murine IL-1β and IL-23 but not IL-6 and IL-12p70. Therefore, OMV secretion is a useful tool of *T. denticola* and *P. gingivalis* for evading host immune systems.

In conclusion, this study demonstrated that BMDCs stimulated with *P. gingivalis* 33277 OMVs and *T. denticola* 33521 OMVs induced the differentiation of Th17 cells and that *T. forsythia* 43037 OMVs favored Th1 cells polarization rather than Th17 cells. IL-6 and IL-12 released from OMV-primed BMDCs played pivotal roles in Th17 and Th1 polarization, respectively. These results suggest that the OMVs of ‘red complex’ periodontal pathogens may play a central role in the pathogenesis of periodontitis by inducing Th1 and Th17 cells through activation of DCs. The limitations of this study were that 1) only one strain of each bacterium was used, 2) OMVs derived from different growth phases and various culture media were not analyzed, 3) only mouse cells, not human cells, were used, and 4) the mechanisms by which OMVs act on the BMDCs remain unsolved. In the future, these issues must be addressed to clarify the role of OMVs of ‘red complex’ periodontal pathogens in T cell differentiation.

## Supplementary Material

Supplemental Material
