# Supplementary material for: Activation of bone marrow-derived dendritic cells and CD4+ T cell differentiation by outer membrane vesicles of periodontal pathogens
Source: J Oral Microbiol. 2022 Sep 14;14(1):2123550. doi: 10.1080/20002297.2022.2123550 (PMC9616074; doi:10.1080/20002297.2022.2123550)
Supplement: Supplemental Material [file ZJOM_A_2123550_SM3230.docx]

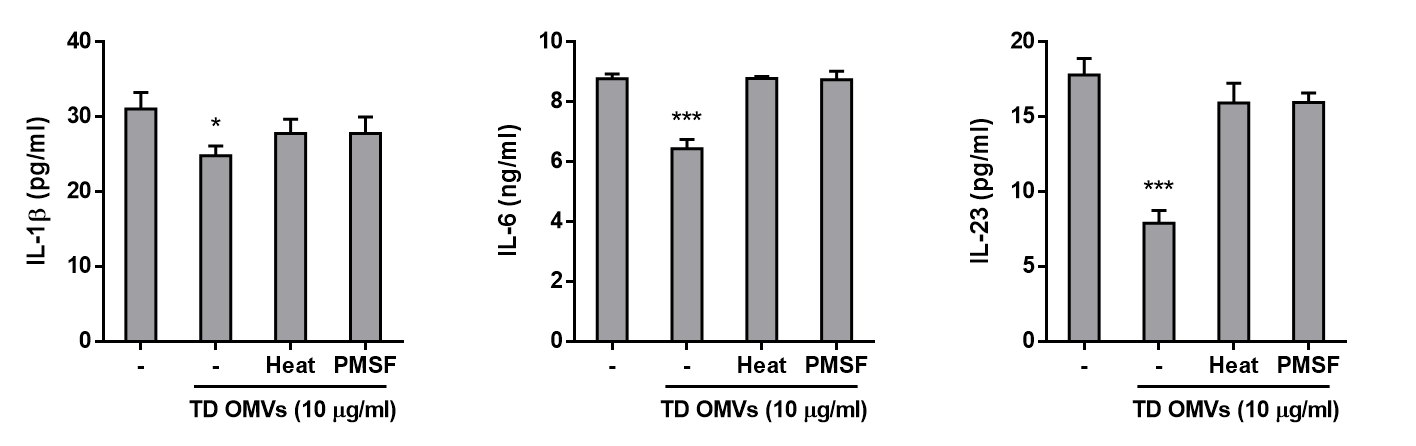


**Supplementary Figure 1. Proteolytic activity of *T. denticola* OMVs against pro-inflammatory cytokines secreted from BMDCs**

BMDCs were treated with Pam3CSK4 (100 ng/ml) for 24 h. The culture supernatants of BMDCs were harvested and then incubated with heat- or PMSF-treated *T. denticola* OMVs for 1 h. The level of the cytokines that remained in the culture supernatants was analyzed using ELISA. The data were presented as means ± SD of triplicate assays and were analyzed by one-way ANOVA. * *p* < 0.05 and *** *p* < 0.001 compared to the control. TD, *T. denticola*.


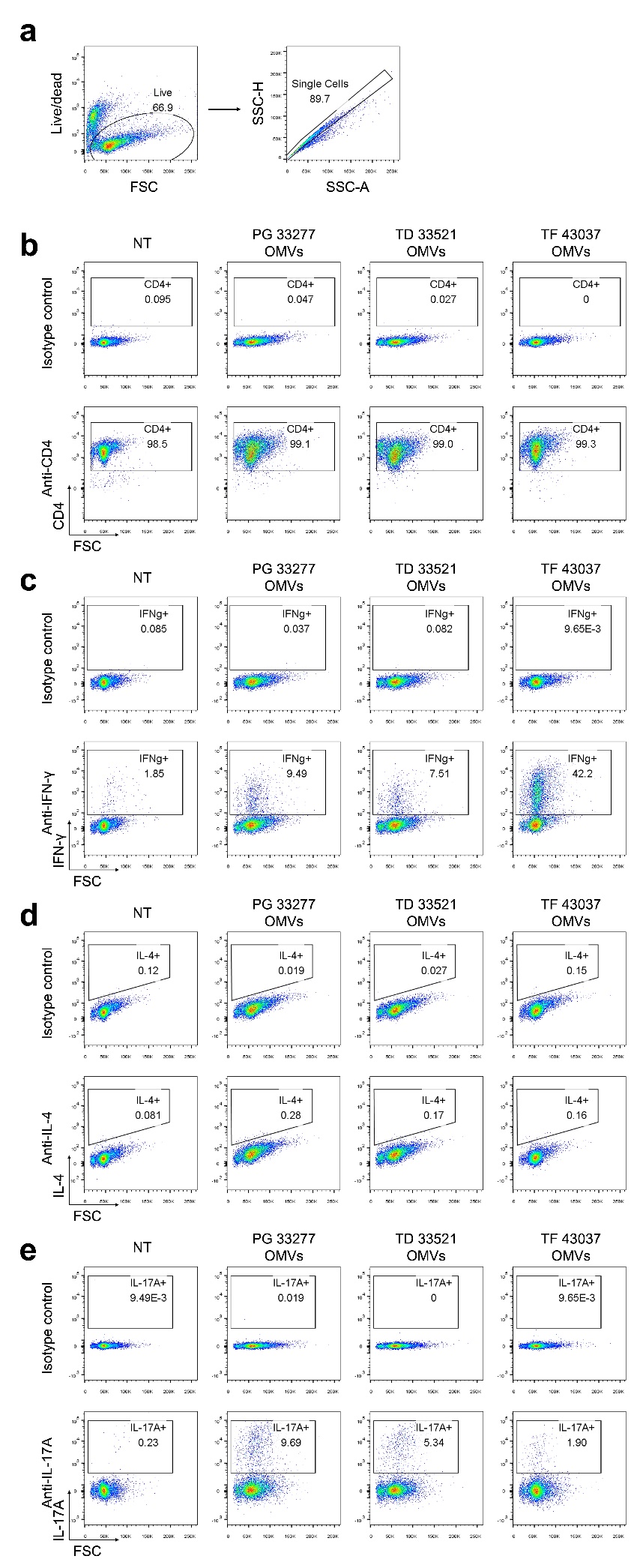


**Supplementary Figure 2. Analysis of isotype control antibody fluorescence in naïve CD4^+^ T cells cocultured with OMV-primed BMDCs**

BMDCs were stimulated with 10 μg/ml of the indicated OMVs for 5 h. After three times of washing with PBS, the BMDCs were cocultured with naïve CD4^+^ T cells for 4 days in the presence of soluble anti-CD3ε antibody and analyzed via flow cytometry. The dead cells were discriminated by live/dead staining and only single cells were gated (a). The fluorescence of anti-CD4 (b), anti-IFN-γ (c), anti-IL-4 (d), and anti-IL-17A (e) and their isotype control antibodies is shown as dot plots. NT, non-treatment; PG, *P. gingivalis*; TD, *T. denticola*; TF, *T. forsythia*; FSC, forward scatter; SSC, side scatter.
